# Circularly polarised luminescence from excimer emission of anthracene derivatives complexed with γ-cyclodextrin in the solid state†

**DOI:** 10.1039/d2ra07971b

**Published:** 2023-01-11

**Authors:** Yuna Kakimoto, Ryoya Ikemura, Yoshitane Imai, Norimitsu Tohnai, Shoko Yamazaki, Eiji Nakata, Hiroshi Takashima

**Affiliations:** a Department of Chemistry, Biology and Environmental Science, Faculty of Science, Nara Women's University Nara 630-8506 Japan hiroshi@cc.nara-wu.ac.jp; b Department of Applied Chemistry, Faculty of Science and Engineering, Kindai University Osaka 577-8502 Japan; c Department of Applied Chemistry, Graduate School of Engineering, Osaka University Osaka 565-0871 Japan; d Department of Chemistry, Nara University of Education Nara 630-8528 Japan; e Institute of Advanced Energy, Kyoto University Kyoto 611-0011 Japan

## Abstract

In this study, we report circularly polarised luminescence (CPL)-active molecules that exhibit high fluorescence quantum yields in the solid state. We developed anthracene derivatives with substituents at the 9 and 10 positions, such as ethyl(anthracene-9-carbonyl)glycinate (9AnGlyEt), *N*-butylanthracene-9-carboxamide (9AnB), *N*-benzylanthracene-9-carboxamide (9AnPh), and *N*^9^,*N*^10^-dibutylanthracene-9,10-dicarboxamide (9,10AnB). These compounds were complexed with γ-cyclodextrin (γ-CD) in the solid state by grinding, and the fluorescence properties of the resulting γ-CD complexes were investigated. The fluorescence quantum yields were enhanced after γ-CD complexation. Among the prepared γ-CD complexes, 9AnGlyEt/γ-CD had the highest fluorescence quantum yield (*Φ*_f_ = 0.35), which was enhanced up to 5.8 times after γ-CD complexation. This was probably due to the interaction between the two anthracene molecules in the γ-CD cavity, which prevented fluorescence quenching caused by aggregation of the compounds. Positive CPL of *g*_CPL_ = 1.3 × 10^−3^ was observed for 9AnGlyEt/γ-CD based on its excimer emission.

## Abstract 2

The 9-substituted anthracene derivative forms a spatially restricted dimer in the cavity of γ-cyclodextrin and exhibits circularly polarised luminescence with *g*_CPL_ = +1.3 × 10^−3^ in the solid state.

## Graphical abstract

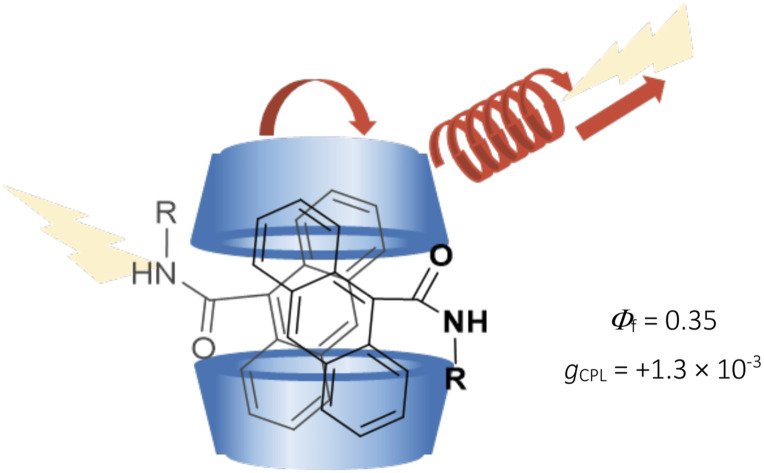

## Introduction

Organic light-emitting dyes that emit circularly polarised luminescence (CPL) have attracted considerable attention and have been actively studied owing to their photophysical behaviour.^1–7^ At present, CPL materials are used in various optoelectronic and biophotonic devices, including polarised light sources for high-brightness liquid crystal displays, 3D displays, paints for advanced security applications, and bioprobes.^8,9^ CPL is important in understanding excited-state chirality and asymmetric photochemistry. To obtain CPL, a quarter-wave plate is used to convert linearly polarised light into circularly polarised light, and right and left circularly polarised light can be obtained; however, the use of filters significantly reduces the light intensity and reduces energy efficiency.^10^ To solve this problem, organic dyes with rigid chiral skeletons that selectively produce positive (+) and negative (−) CPL have been designed.^11,12^ However, designing such chiral chromophores is often difficult because of the limited availability of materials, multi-step synthesis processes, and time-consuming chiral separation processes.

Pyrene derivatives form a 2 : 2 complex with γ-cyclodextrin (γ-CD) in water, and the two molecules are encapsulated in a twisted state in the cavity.^13^ Previously, we demonstrated CPL from achiral hydrophobic pyrene derivatives and γ-CD complexes in water.^14^ The pyrene and γ-CD complexes were prepared by grinding in the solid state. Using this approach, CPL-active substances can be obtained without optical resolution or multi-step synthesis of organic dyes because the γ-CD asymmetry is exploited. These spatially restricted excimer-derived CPL-active complexes exhibit efficient CPL properties.^15^ Anthracene, which is similar to pyrene, also exhibits excimer fluorescence in the solid state, and its colour varies depending on the degree of overlap of the anthracene rings.^16–20^

Organic electronic luminescent materials are often used in the solid state.^21–23^ Although effective CPL materials require high asymmetry coefficient |*g*_lum_| values and high fluorescence quantum yields (*Φ*_f_), there are few reported cases of CPL observations in the solid state.^24,25^ This is due to the decrease in the fluorescence quantum yield caused by aggregation of the compounds in the solid state.^26,27^ Ema and co-workers reported CPL in the solid state of compounds utilising the large steric hindrance of axially chiral compounds and the aggregation-induced emission mechanism.^24,25^ However, the fluorescence quantum yields of these compounds are low (approximately 0.2). To improve the fluorescence quantum yield, fluorescent molecules incorporated into γ-CD in the solid state can be spatially controlled in terms of the number of molecules, molecular orientation, and degree of overlap of π-planes, which prevents the deactivation of the excitation energy of fluorescent molecules.^28^ Therefore, inclusion in γ-CD in the solid state not only induces chirality in the fluorescent molecule but also improves its quantum yield.

**Fig. 1:**
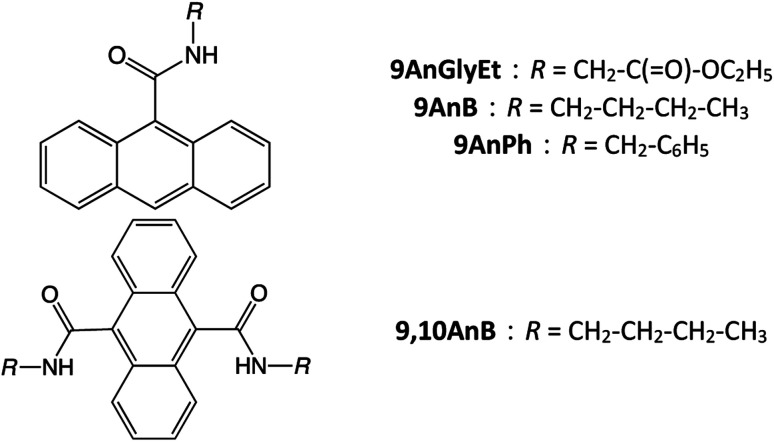
Chemical structures of 9AnGlyEt, 9AnB, 9AnPh, and 9,10AnB.

In this study, we investigated anthracene derivatives with substituents at the 9 and/or 10 positions, such as ethyl(anthracene-9-carbonyl)glycinate (9AnGlyEt), *N*-butylanthracene-9-carboxamide (9AnB), *N*-benzylanthracene-9-carboxamide (9AnPh), and *N*^9^,*N*^10^-dibutylanthracene-9,10-dicarboxamide (9,10AnB) (Fig. 1). In the case of anthracene derivatives with substituents at the 2 position, the dimerization reaction is known to proceed readily in the γ-CD cavity under light irradiation.^29^ As such dimers do not show excimer fluorescence, we considered 9-substituted anthracene derivatives to design the compounds in this study. Furthermore, the introduction of an amide bond in the substituent enables hydrogen bonding with the hydroxyl groups of γ-CD. We also developed three derivatives with different steric hindrances by varying the alkyl and aromatic substituents. The fluorescence properties of the new γ-CD complexes, prepared by mixing and grinding these compounds with γ-CD in the solid state, were investigated experimentally and theoretically. Density functional theory (DFT) calculations were used for the theoretical investigation of the fluorescence properties. Furthermore, the CPL properties of the prepared complexes and their inclusion mechanism were studied.

## Results and discussion

### Synthesis of anthracene derivatives

9AnGlyEt, 9AnB, and 9AnPh were synthesised by condensation of glycine ethyl ester hydrochloride, *n*-butylamine, and benzylamine, respectively, with 9-anthracenecarboxylic acid. 9,10AnB was synthesised by condensation of *n*-butylamine and 9,10-anthracenedicarboxylic acid. The details of the synthesis and identification of the compounds are provided in the ESI (Scheme S1, Fig. S1–S7, and Tables S1–S3†).

### Crystal structures of anthracene derivatives

**Fig. 2:**
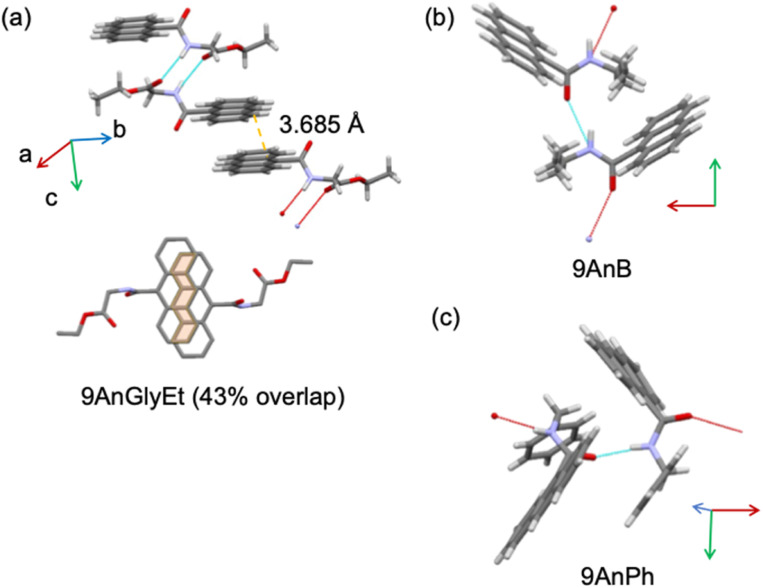
Crystal structures of (a) 9AnGlyEt, (b) 9AnB, and (c) 9AnPh, as visualized using the Mercury software.

The crystal structures of the substituted anthracene derivatives were determined using X-ray crystallography. The three-dimensional representations of 9AnGlyEt, 9AnB, and 9AnPh obtained using the Mercury visualisation tool are shown in Fig. 2, and the corresponding crystallographic data are presented in Table 1.

**Table 1:** Crystallographic data for 9AnGlyEt, 9AnB, and 9AnPh

	9AnGlyEt	9AnB	9AnPh
Chemical formula	C_19_H_17_NO_3_	C_19_H_19_NO	C_22_H_17_NO
Formula weight	307.35	277.37	311.37
Crystal system	Triclinic	Orthorhombic	Triclinic
Space group	*P*1̄	*Pbca*	*P*1̄
*a* (Å)	8.9450	9.74760(16)	9.0368(5)
*b* (Å)	9.2434	9.04653(15)	15.2490(7)
*c* (Å)	10.47923	34.1643(7)	24.7924(6)
*α* (deg)	111.862	90.0000	90.484
*β* (deg)	93.627	90.0000	93.805
*γ* (deg)	103.670	90.0000	90.777
*Z*	2	8	2
*λ* (Mo Kα)	1.54187	1.54184	1.54184
*θ* max	73.91	74.250	73.5730
*μ* (cm^−1^)	7.29	5.84	0.579
*D*_c_ (mg m^−3^)	1.078	1.223	1.214
*T* (K)	296	123	123
*V* (Å^3^)	770.29	3012.67(9)	3408.5(2)
*R*_1_	0.0529	0.0410	0.1618
*R*_w_	0.1573	0.1126	0.4055
GoF	1.377	1.046	2.604

Crystal structure analysis of 9AnGlyEt showed that intermolecular hydrogen bonds were formed between the ester and amide groups, and the anthracene moieties overlapped with an estimated area of 43% (Fig. 2a). In contrast, 9AnB and 9AnPh, which do not have an ester group, showed intermolecular hydrogen bond formation at the amide group but no overlap of the anthracene moieties was observed (Fig. 2b and c).

### Preparation of anthracene derivative/γ-CD complexes

Anthracene derivative/γ-CD complexes were prepared using a previously reported method.^30^ The anthracene derivatives (4.5 × 10^−6^ mol) and γ-CD (4.5 × 10^−6^ mol) were mixed in the solid state, followed by grinding for 30 min using a mortar until the colour of the solid became constant. Chloroform was added to the mixture, which was then stirred for 1 h and filtered through a membrane filter, and the residue was washed with chloroform. The resulting residue was vacuum dried for 5 h to obtain a composite sample. In this study, chloroform was added to eliminate the effect of unincorporated anthracene on the fluorescence and other photophysical properties of the anthracene derivative/γ-CD complexes, as washing with chloroform removed unincorporated anthracene molecules.

### Fluorescence properties of anthracene derivatives and γ-CD complexes

**Fig. 3:**
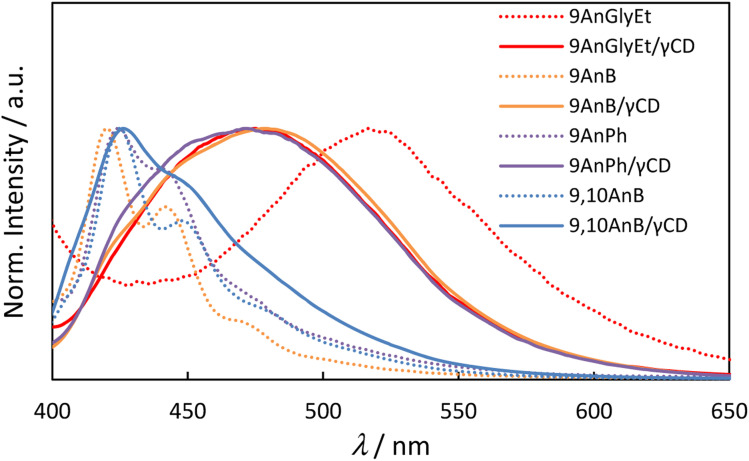
Fluorescence spectra of anthracene derivatives (9AnGlyEt, 9AnB, 9AnPh, and 9,10AnB) and their γ-CD complexes (9AnGlyEt/γ-CD, 9AnB/γ-CD, 9AnPh/γ-CD, and 9,10AnB/γ-CD) in the solid state (*λ*_ex_ = 370 nm).

The fluorescence spectra of the anthracene derivatives before and after inclusion in γ-CD are presented in Fig. 3 and the corresponding fluorescence properties are summarised in Table 2. As expected from the crystal structures of the compounds, 9AnGlyEt, in which the anthracene moieties overlapped, exhibited excimer fluorescence, whereas 9AnB and 9AnPh exhibited monomer fluorescence. After γ-CD inclusion, the fluorescence quantum yields of all the compounds were significantly enhanced. In particular, the formation of the 9AnGlyEt/γ-CD complex resulted in a 5.8-fold increase in the fluorescence quantum yield (*Φ*_f_ = 0.35). Inclusion in γ-CD may prevent quenching-derived aggregation of the compounds. Furthermore, the fluorescence wavelengths of 9AnGlyEt/γ-CD was blue-shifted (Δ*λ* = −42 nm; Table 2), whereas those of 9AnB/γ-CD and 9AnPh/γ-CD were red-shifted (Δ*λ* = 58 and 47 nm, respectively; Table 2) compared to those before inclusion. These shifts are probably due to changes in the overlap of the anthracene moieties of the two molecules. This observation is consistent with the results of a previous study, which revealed that greater overlap of the anthracene moieties causes longer wavelength emission.^16^ The shift to longer wavelengths was observed as a change in the colour of the solid-state emission in the visible region (Fig. S8†).

**Table 2:** Fluorescence properties of anthracene derivatives and their γ-CD complexes in the solid state

Complexes or compounds	*λ*_ex_/nm	*λ*_em_/nm	*Φ*_f_ a	Total shift^b^ Δ*λ*/nm
9AnGlyEt	370	517	0.06	−42
9AnGlyEt/γ-CD	370	475	0.35	
9AnB	370	420	0.16	58
9AnB/γ-CD	370	478	0.27	
9AnPh	370	424	0.11	47
9AnPh/γ-CD	370	471	0.34	
9,10AnB	370	425	0.21	1
9,10AnB/γ-CD	370	426	0.26	

a Emission quantum yield was calculated as the absolute quantum yield.

b Total shift Δ*λ* = *λ*_em,complex_ − *λ*_em,anthracene_.

The fluorescence spectra of 9,10AnB exhibited small changes (1 nm) before and after inclusion in γ-CD. Moreover, only monomer fluorescence was observed, which suggests that only one molecule of 9,10AnB is encapsulated within the γ-CD cavity, and the anthracene moieties do not overlap easily because of steric repulsion between the substituents at the 9 and 10 positions.

### Emission lifetimes of anthracene derivatives and γ-CD complexes

**Fig. 4:**
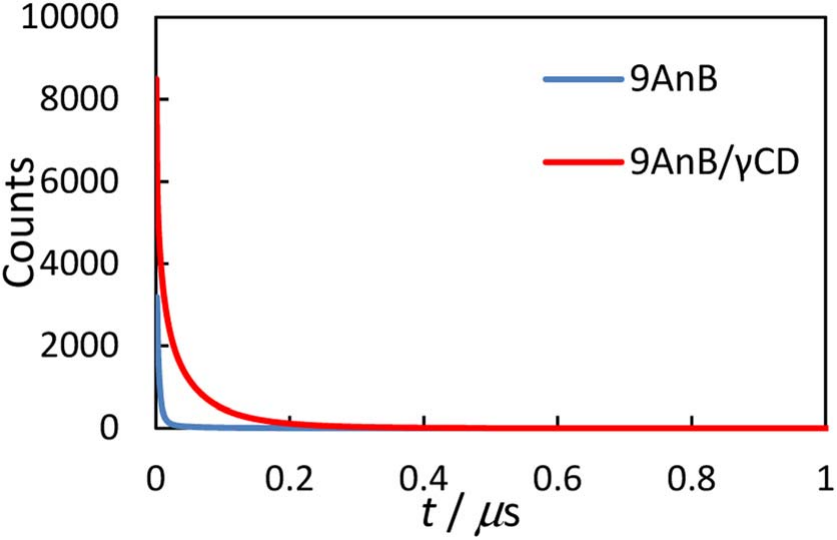
Emission decay curves of 9AnB (blue line) and 9AnB/γ-CD (red line) in the solid state at room temperature (*λ*_ex_ = 370 nm, *λ*_em_ > 420 nm).

Next, we measured the solid-state emission lifetimes of the 9-substituted anthracene derivatives and their γ-CD complexes (Fig. 4). The results are summarised in Table 3. The decay profiles were analysed using the following equation:*I*_*t*_ = *A*_1_ exp(−*t*/*τ*_1_) + *A*_2_ exp(−*t*/*τ*_2_) + *A*_3_ exp(−*t*/*τ*_3_) + *A*_4_ exp(−*t*/*τ*_4_)where *I*_*t*_ is the emission intensity, *t* is the time, *τ* is the lifetime, and *A* is the fractional contribution.

**Table 3:** Emission lifetimes of anthracene derivatives and their γ-CD complexes in the solid state at room temperature (*λ*_ex_ = 370 nm, *λ*_em_ > 420 nm)

Complexes or compounds	Emission lifetime/ns (*A*/%)					*χ* ^2^
	*τ*_1_	*τ*_2_	*τ*_3_	*τ*_4_	*τ*_ave_	
9AnGlyEt	6.15 (5)	26.3 (84)	65.6 (12)		30.3	1.08
9AnGlyEt/γ-CD	1.41 (2)	10.3 (11)	41.9 (48)	98.2 (38)	58.6	1.02
9AnB	2.46 (38)	6.12 (39)	38.6 (23)		12.2	0.90
9AnB/γ-CD	1.20 (2)	8.98 (12)	36.0 (40)	84.6 (46)	54.4	1.03
9AnPh	2.12 (57)	6.77 (24)	41.8 (19)		10.8	1.10
9AnPh/γ-CD	1.20 (2)	8.82 (14)	36.9 (47)	94.4 (37)	53.5	1.08

An excimer-fluorescence-derived long-lived component (*τ*_4_) was observed for the γ-CD complexes. This lifetime is comparable to previously reported values for the anthracene excimer in the solid state (59–135 ns).^16^ In addition, longer fluorescence lifetimes (*τ*_ave_) were observed for all the complexes. These results are consistent with the increase in the fluorescence quantum yield, as the extended lifetime is possibly due to γ-CD preventing quenching through intermolecular energy deactivation. The two anthracene molecules may form a complex with γ-CD in the solid state, thereby preventing the molecules from aggregating.

### Diffuse reflectance spectra of anthracene derivatives and γ-CD complexes

**Fig. 5:**
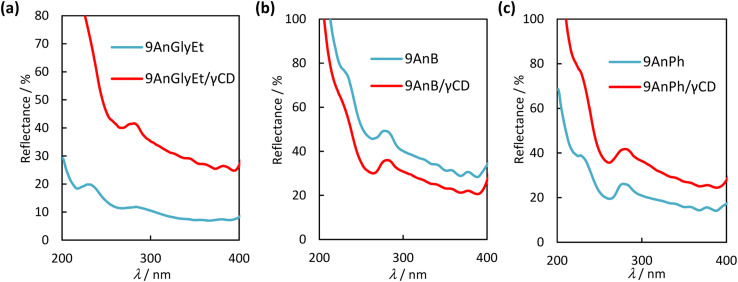
Diffuse reflectance spectra of (a) 9AnGlyEt (blue line) and 9AnPh/γ-CD (red line), (b) 9AnB (blue line) and 9AnB/γ-CD (red line), and (c) 9AnPh (blue line) and 9AnPh/γ-CD (red line) in the solid state.

Diffuse reflectance spectra of the 9-substituted anthracene derivatives were measured to investigate the interaction of the π-planes in the ground state before and after complexation with γ-CD. The spectra of 9AnGlyEt, 9AnB, 9AnPh, and their γ-CD complexes in the solid state are shown in Fig. 5. The observed absorption peaks are presented in Table 4. In general, a large overlap of planes results in band broadening because of intermolecular interactions. As expected from the crystal structure, 9AnGlyEt, with overlapping anthracene moieties, exhibited a broadened peak, whereas 9AnB and 9AnPh exhibited peaks with a clear vibrational structure. After γ-CD inclusion, a peak with a loose vibrational structure was observed for all three complexes, as shown by the red line in Fig. 5. These results are consistent with the observation of excimer fluorescence in the emission spectra and lifetimes, suggesting that inclusion in γ-CD affected the molecular arrangement of anthracene, resulting in overlapping anthracene rings.

**Table 4:** Diffuse reflectance spectral data for anthracene derivatives and their γ-CD complexes in the solid state

Complexes or compounds	*λ*_abs_/nm
9AnGlyEt	284, 357, 382
9AnGlyEt/γ-CD	281, 357, 381
9AnB	277, 357, 377
9AnB/γ-CD	281, 355, 377
9AnPh	278, 356, 377
9AnPh/γ-CD	281, 355, 377

### DFT and time-dependent DFT calculations for the ground and excited states of anthracene derivatives

**Fig. 6:**
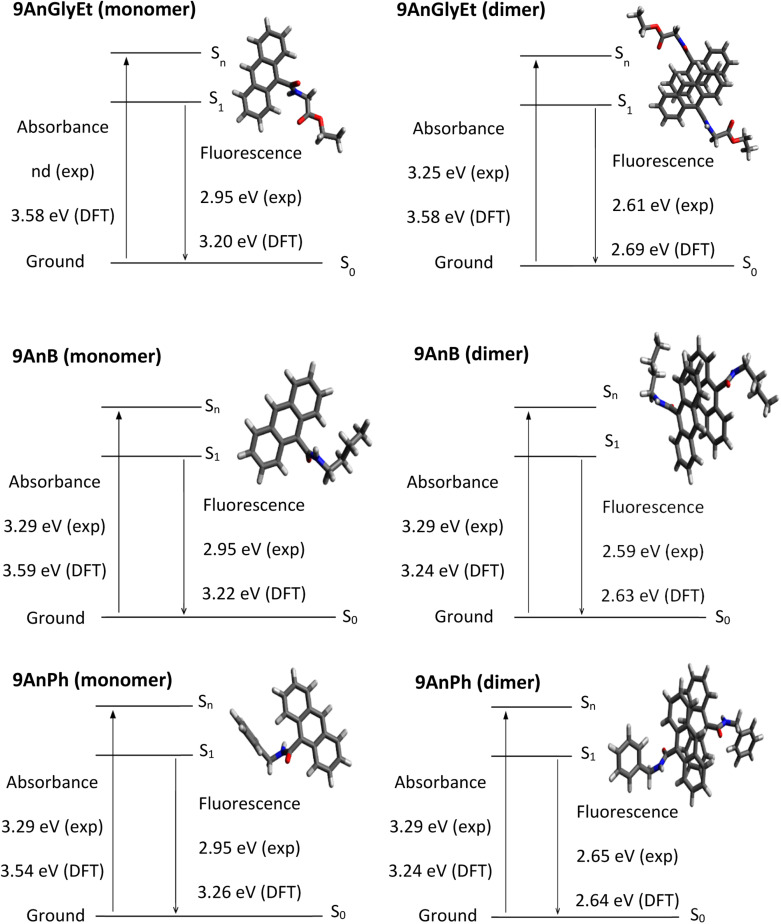
Energy diagrams for 9AnGlyEt (monomer), 9AnGlyEt (dimer), 9AnB (monomer), 9AnB (dimer), 9AnPh (monomer), and 9AnPh (dimer).

Theoretical analyses of the monomers and dimers of the anthracene derivatives were performed using DFT calculations with the Gaussian 16 programme package^31^ to obtain energy diagrams (eV) in the ground and singlet excited states. The ωB97X-D functional^32^ with the 6-31+G* basis set was used to calculate 9AnGlyEt. In the cases of 9AnB and 9AnPh, the M06-2X functional^33^ and the 6-31+G* basis set were used. The calculated initial structures of 9AnGlyEt, 9AnB, and 9AnPh were determined using the data obtained from X-ray crystallography. Time-dependent DFT (TD-DFT) calculates were used to compare the ground state and singlet excited state geometries. The ground state and singlet excited state energies, corresponding to the absorption and fluorescence wavelengths, respectively, obtained from the DFT calculations of the anthracene monomers and dimers are shown in Fig. 6. The experimental values of the absorption energies of the anthracene compounds were obtained from the diffuse reflectance spectra. The calculated fluorescence energies of 9AnGlyEt (dimer), 9AnB (dimer), and 9AnPh (dimer) were 2.69, 2.63, and 2.64 eV, respectively, which are in good agreement with those of the corresponding anthracene/γ-CD complexes in the solid state (2.61 eV, 2.59 eV, and 2.65 eV, respectively). These results suggest that anthracene molecules form dimers in the γ-CD cavity, even when they form inclusion complexes.

### Circular dichroism and CPL spectral properties of anthracene derivatives with γ-CD complexes

**Fig. 7:**
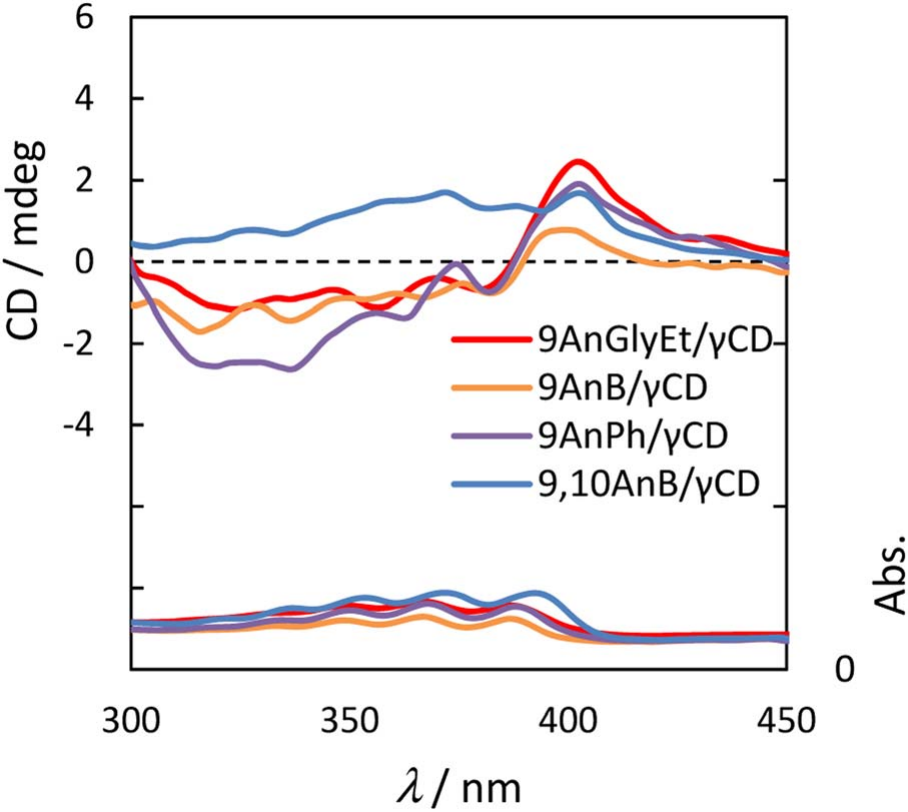
Circular dichroism (upper panel) and UV spectra (lower panel) of 9AnGlyEt/γ-CD (red line), 9AnB/γ-CD (green line), 9AnPh/γ-CD (yellow line), and 9,10AnB/γ-CD (blue line) in KBr pellets.

Because γ-CD is a chiral molecule, anthracene molecules in the γ-CD cavity are in a chiral environment in the ground state. Therefore, we measured the circular dichroism spectra of 9AnGlyEt/γ-CD, 9AnB/γ-CD, 9AnPh/γ-CD, and 9,10AnB/γ-CD. The measurements were performed using the KBr tablet method, and the results are presented in Fig. 7 and Table 5.

**Table 5:** Circular dichroism spectral data for 9AnGlyEt/γ-CD, 9AnB/γ-CD, 9AnPh/γ-CD, and 9,10AnB/γ-CD in KBr pellets

Complexes	*λ*_1_/nm (sign)	*λ*_2_/nm (sign)
9AnGlyEt/γ-CD	388 (−)	404 (+)
9AnB/γ-CD	391 (−)	404 (+)
9AnPh/γ-CD	390 (−)	408 (+)
9,10AnB/γ-CD	372 (+)	—

The anthracene/γ-CD complexes showed a strong Cotton effect in the anthracene absorption region from 300 to 450 nm. For 9AnGlyEt/γ-CD, 9AnB/γ-CD, and 9AnPh/γ-CD, a positive first Cotton effect and a negative second Cotton effect were observed at approximately 404 and 390 nm, respectively, in the longer wavelength region. According to the exciton chirality method, the circular dichroism spectrum in the longer wavelength region exhibits a positive-to-negative Cotton effect when two chromophores with right-handed rotation are located close to each other.^34^ Thus, the 9-substituted anthracene molecules are anticipated to interact with the γ-CD cavity in the ground state and twist with right-handed chirality. However, only a positive Cotton effect was observed in the anthracene absorption region for 9,10AnB/γ-CD and no split Cotton effect was observed. This suggests single-molecule inclusion in the γ-CD cavity of the 9,10AnB/γ-CD complex, which is consistent with the experimental observation of monomer fluorescence (Fig. 3).

We used α-CD and β-CD, which have different cavity sizes, to examine the effect of cyclodextrin size on the circular dichroism spectra, and the results for 9AnGlyEt/α-CD, 9AnGlyEt/β-CD, and 9AnGlyEt/γ-CD are shown in Fig. S9.† With α-CD and β-CD, no strong Cotton effect appeared in the anthracene absorption region, as observed for 9AnGlyEt/γ-CD. Thus, the split Cotton effect observed for the 9-substituted anthracene/γ-CD complexes likely originates from the inclusion of two anthracene molecules in the γ-CD cavity.

**Fig. 8:**
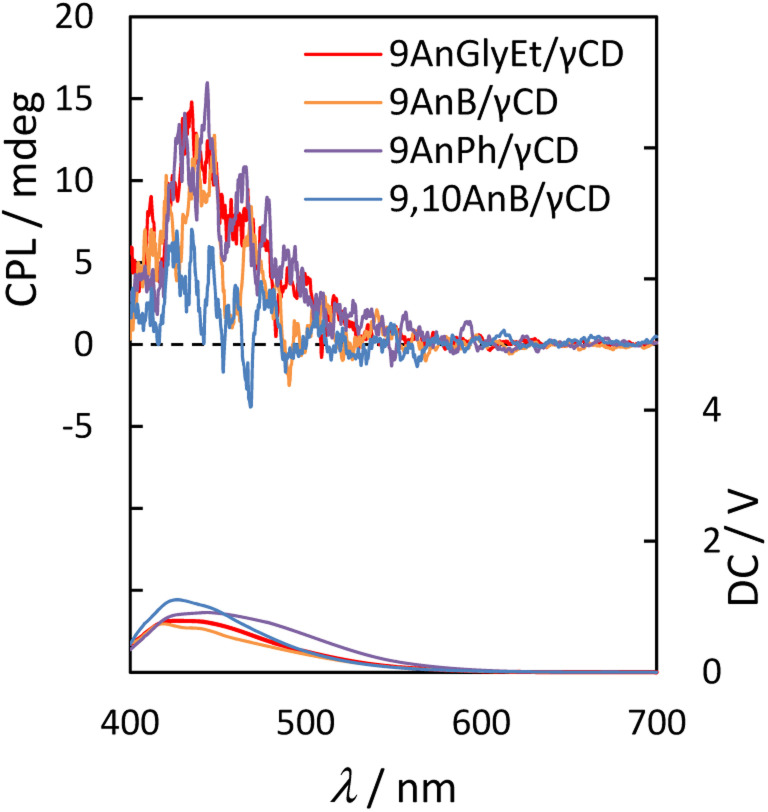
CPL (upper panel) and photoluminescence (lower panel) spectra of 9AnGlyEt/γ-CD (red line), 9AnB/γ-CD (green line), 9AnPh/γ-CD (yellow line), and 9,10AnB/γ-CD (blue line) in KBr pellets (*λ*_ex_ = 370 nm).

Next, we measured the CPL spectra of 9AnGlyEt/γ-CD, 9AnB/γ-CD, 9AnPh/γ-CD, and 9,10AnB/γ-CD, which exhibited chirality in the ground state, using the KBr pellet method. To evaluate the CPL characteristics quantitatively, we used the dimensionless Kuhn's asymmetry factor, which is defined as*g*_lum_ = (*I*_L_ − *I*_R_)/[1/2(*I*_L_ + *I*_R_)]where *I*_L_ and *I*_R_ are the emission intensities of the leftward and rightward circularly polarised components under unpolarised excitation, respectively. Fig. 8 and Table 6 show the CPL spectra and properties, respectively. 9AnGlyEt/γ-CD and 9AnB/γ-CD exhibited positive CPL with an asymmetry factor *g*_lum_ of +1.3 × 10^−3^. For 9AnPh/γ-CD, positive CPL with *g*_lum_ = +1.2 × 10^−3^ was observed. The asymmetry factor values of these anthracene derivative/γ-CD complexes are comparable (∼10^−3^) to those of pyrene/γ-CD complexes in water reported previously.^14,35^ The signs of all the CPL signals for the 9-substituted anthracenes were consistent with the first Cotton effect in the circular dichroism spectra (Fig. 7). Thus, in the excited state, all the 9-substituted compounds may interact with the γ-CD cavity and be twisted with right-handed chirality, similar to the behaviour in the ground state.

**Table 6:** CPL properties of 9AnGlyEt/γ-CD, 9AnB/γ-CD, 9AnPh/γ-CD, and 9,10AnB/γ-CD in KBr pellets (*λ*_ex_ = 370 nm)

Complexes	*λ*_ex_/nm	*λ*_CPL_/nm	*g*_lum_ (× 10^−3^)
9AnGlyEt/γ-CD	370	435	+1.3
9AnB/γ-CD	370	438	+1.3
9AnPh/γ-CD	370	444	+1.2
9,10AnB/γ-CD	370	435	+0.46

However, weak CPL with *g*_lum_ = +0.46 × 10^−3^ was observed for 9,10AnB/γ-CD, which was expected to have single-molecule inclusion based on the fluorescence and CD spectra. The *g*_lum_ value of 9,10AnB/γ-CD was considerably lower than that of 9AnB/γ-CD. These results indicate that the inclusion of two anthracene molecules in γ-CD is effective for CPL.

### Interactions between anthracene derivatives and γ-CD

**Fig. 9:**
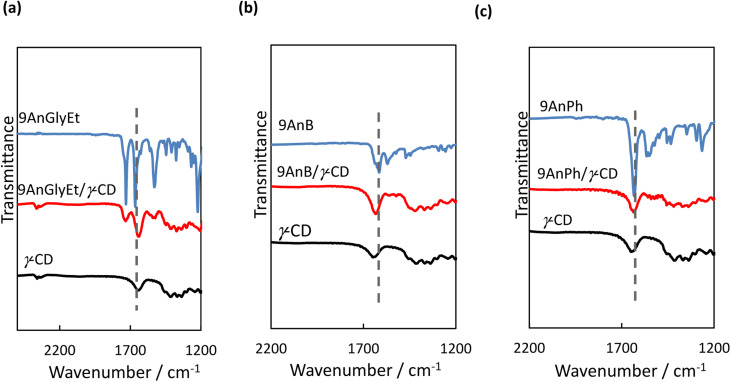
FT-IR spectra of (a) 9AnGlyEt, 9AnGlyEt/γ-CD, and γ-CD; (b) 9AnB, 9AnB/γ-CD, and γ-CD; and (c) 9AnPh, 9AnPh/γ-CD, and γ-CD in KBr pellets.

Fourier transform infrared (FT-IR) spectroscopy measurements were performed to investigate the interactions between the anthracene derivatives and γ-CD. All measurements of 9AnGlyEt, 9AnB, 9AnPh, and their γ-CD complexes were performed using the KBr pellet method, and the obtained FT-IR spectra are shown in Fig. 9. The spectra exhibited characteristic peaks corresponding to C=O stretching around 1730 cm^−1^ (ester), C=O stretching around 1640 cm^−1^ (amide), and N–H vibrations around 1540 cm^−1^ (amide). The wavenumber values before and after inclusion in γ-CD are summarised in Table 7. For the 9-substituted anthracene derivatives, a peak shift was observed upon γ-CD inclusion, suggesting non-covalent interactions with γ-CD. Focusing on the C=O stretching of the amide group, a significant shift (27 cm^−1^) to a lower wavenumber was observed for 9AnGlyEt after γ-CD inclusion, presumably due to the formation of hydrogen bonds with γ-CD. In contrast, the C=O stretching peaks for 9AnB and 9AnPh were observed at 1615 and 1633 cm^−1^, respectively, owing to the formation of intermolecular hydrogen bonds in the original solid state (Fig. 2). After complex formation with γ-CD, the peak corresponding to C=O stretching in the amide shifted to higher wavenumbers. The amide C=O stretching peaks of the three complexes were located at similar wavenumbers, suggesting that the amide C=O groups were in the same environment. This could be due to the formation of hydrogen bonds between the amide C=O group and a hydroxyl group of γ-CD.

**Table 7:** Wavenumbers of selected FT-IR peaks for anthracene derivatives and their γ-CD complexes

Complexes	Wavenumber/cm^−1^		
	C=O stretching (ester)	C=O stretching (amide)	N–H angular (amide)
9AnGlyEt/γ-CD	1730 → 1732	1665 → 1638	1529 → 1541
9AnB/γ-CD	—	1615 → 1634	1569 → 1559, 1541
9AnPh/γ-CD	—	1633 → 1636	1564, 1550 → 1540

In addition, solid-state ^13^C cross-polarisation/magic-angle spinning (CP/MAS) NMR measurements of 9AnGlyEt, γ-CD, and 9AnGlyEt/γ-CD were performed to investigate the interactions between the anthracene derivatives and γ-CD in the solid state. The spectra are shown in Fig. S10–S13† and the corresponding properties are summarised in Tables S4–S6.† The identification of 9AnGlyEt and γ-CD signals was based on previously reported ^13^C NMR spectral data in solution and the solid state.^36^ Before mixing with γ-CD, 9AnGlyEt showed sharp peaks; however, after mixing, a broadened peak was observed. This indicates an interaction between 9AnGlyEt and γ-CD and that the complex is in an amorphous state. γ-CD without guest molecules exhibited low symmetry and split peaks; however, broadened peaks appeared after complex formation. Similar peaks were observed in previously reported solid-state ^13^C NMR spectra of γ-CD/aromatic compound complexes.^37,38^ These results suggest that the complex is in an amorphous state with guest molecules encapsulated within γ-CD. Because the anthracene derivative is insoluble in water, it was difficult to determine the stoichiometric ratio of the complexes using NMR measurements in aqueous solution. Owing to these experimental limitations, we currently assume that the stoichiometry of the anthracene/γ-CD complex is 2 : 2.

## Conclusions

In this study, we developed CPL-active molecules that exhibit high fluorescence quantum yields in the solid state. Anthracene derivatives substituted at the 9 and 10 positions and their complexes with γ-CD were prepared, and their fluorescence properties were evaluated. The fluorescence quantum yields of the anthracene derivates were enhanced after interaction with γ-CD. Among them, 9AnGlyEt/γ-CD exhibited the highest fluorescence quantum yield (*Φ*_f_) of 0.35, which was up to 5.8 times higher than that of 9AnGlyEt. This enhancement is attributed to the inclusion of two anthracene molecules in the γ-CD cavity, which prevents quenching due to aggregation of the compounds. Positive CPL with an asymmetry factor *g*_lum_ of 1.3 × 10^−3^ was observed for the 9-substituted anthracenes that exhibited excimer fluorescence. This asymmetry factor value is comparable (∼10^−3^) to those of pyrene/γ-CD complexes in water reported previously.^12,32^ Furthermore, hydrogen bonding interactions between the amide C=O group of the anthracene derivative and the hydroxyl group of γ-CD are considered important for complex formation.

## Author contributions

Yuna Kakimoto: data curation, formal analysis, visualization. Ryoya Ikemura: investigation. Yoshitane Imai: resources. Norimistu Tohnai: resources. Shoko Yamazaki: investigation. Eiji Nakata: resources. Hiroshi Takashima: conceptualization, funding acquisition, resources, supervision, writing – original draft, writing – review & editing.

## Conflicts of interest

The authors declare no conflict of interest.

## Supplementary Material

RA-013-D2RA07971B-s001

RA-013-D2RA07971B-s002
