# Supplementary material for: Circularly polarised luminescence from excimer emission of anthracene derivatives complexed with γ-cyclodextrin in the solid state
Source: RSC Adv. 2023 Jan 11;13(3):1914–22. doi: 10.1039/d2ra07971b (PMC9832359; doi:10.1039/d2ra07971b)
Supplement: RA-013-D2RA07971B-s001 [file RA-013-D2RA07971B-s001.pdf]

Electronic Supplementary Material (ESI) for RSC Advances.
This journal is © The Royal Society of Chemistry 2023

Electronic Supplementary Information

Circularly polarised luminescence from excimer emission of anthracene derivatives complexed with γ-cyclodextrin in the solid state

Yuna Kamimoto[1], Ryoya Ikemura[2], Yoshitane Imai[2], Norimitsu Tohnai[3], Shoko Yamazaki[4], Eiji Nakata[5], Hiroshi Takashima,[1,*]

[1]Department of Chemistry, Biology and Environmental Science, Faculty of Science, Nara Women's University, Nara 630-8506, Japan

[2] Department of Applied Chemistry, Faculty of Science and Engineering, Kindai University, Osaka 577-8502, Japan

[3] Department of Applied Chemistry, Graduate School of Engineering, Osaka University, Osaka 565-0871, Japan

[4] Department of Chemistry, Nara University of Education, Nara 630-8528, Japan

[5] Institute of Advanced Energy, Kyoto University, Kyoto 611-0011, Japan

***Correspondence**

H. Takashima

Department of Chemistry, Biology and Environmental Science, Faculty of Science,

Nara Women's University, Nara 630-8506, Japan

E-mail: hiroshi@cc.nara-wu.ac.jp

Table of contents

1. Experimental materials

2. Instrumentation

3. Crystallographic data collection and structure determination

4. Computational methods

5. Synthetic methods and characterization

6. Fluorescent color change

7. Circular dichroism spectra

8. ^{13}C CP/MAS NMR

9. Reference

1. Experimental materials

9-Anthracenecarboxylic acid was from Tokyo Kasei Kogyo Co. 9,10-Anthracenedicarboxylic acid and glycine ethyl hydrochloride were from Sigma-Aldrich. 3,3-Dimethylbutylamine, *n*-butylamine, dehydrated *N*,*N*-dimethylformamide (DMF), BOP reagent, γ-cyclodextrin (CD), benzylamine, dichloromethane (DCM), methanol (MeOH), acetone, hexane, chloroform, anhydrous sodium sulfate, sodium hydroxide (NaOH) and Wako Gel C-200 from Fuji Film Wako Pure Chemical Co. were used. KBr was used for IR absorption measurement by Fujifilm Wako Pure Chemicals Co. CD_3OD and $CDCl_3$ for ^1H NMR spectra and ^{13}C NMR were used by Fujifilm Wako Pure Chemicals Co. For ESI-MS measurements, methanol for LC/MS from Fujifilm Wako Pure Chemicals Co. was used. Organic membrane filter PTFE (0.5 μm, 25 mm) was used from ADVANTEC.

2. Instrumentation

ESI-Mass spectra were measured with JEOL IMS-T100LC AccuTOF. ^1H NMR spectra (400 MHz) and ^{13}C NMR (100 MHz) in solution were recorded on a JEOL JNM-AL400 FT-NMR. ^1H NMR chemical shift values are reported in ppm as reference to the internal standard TMS. Elemental analysis was performed on a J-Science Lab MICRO CORDER JM10 and a Yanaco CHN corder MT-5. Melting point was measured on a Yanaco MP-J3 micro melting point apparatus. Fluorescence spectra and the absolute quantum yields were measured by JASCO FP-6600 fluorescence spectrophotometer with an integrating sphere. Time-resolved emission spectra were measured by a single-photon counting method using a Horiba-Jobin Yvon Tempro equipped with a 420 nm cut filter. The instrumental response of the system to the excitation pulsed solid-state LED light source of 370 nm had a time width of about 100 ps and repetition rate about 1 MHz. The lifetimes were evaluated with the software attached to this equipment. CD spectra were measured with Jasco J-720 spectrometers using a KBr pellet. CPL spectra were recorded on a JASCO CPL-300 spectrometer using a KBr pellet. X-ray crystallographic data were obtained by Rigaku R-Axis-Rapid Imaging Plate diffractometer. Fourier transform IR spectra (FT/IR) were measured by the KBr tablet method using the JASCO FT/IR-6100V spectrophotometer. Diffuse reflectance spectra were measured using a Shimadzu UV-2600i with barium sulfate as a standard white plate. Solid-state ^{13}C CP/MAS (Cross-

Polarization/Magic-Angle Spinning) NMR was measured using JEOL JNM-ECZ400R. A 4 mm HXMAS VT probe was used with the CH signal (29.5 ppm) of adamantane as an external reference (rotational speed 15 kHz, room temperature, flip angle 90°). CP/MAS was performed using the TOtal Spinning Sideband (TOSS) method to eliminate spinning sidebands.

3. Crystallographic data collection and structure determination

A crystal was mounted in a loop. All measurements were made on a diffractometer using multi-layer mirror monochromated Cu-Kα or Mo-Kα radiation. The data were collected at a temperature of 23 ± 1 or -150 ± 1 °C using the ω–2θ scan technique to a maximum 2θ value of 67.7° or 152.6°. Data were collected and processed using CrysAlisPro (Rigaku Oxford Diffraction). The structures were solved by direct methods and refined on F^2 by full-matrix least-squares methods, using SHELXL-97 [S1]. The non-hydrogen atoms were refined anisotropically by the full-matrix least-squares method. All hydrogen atoms were isotropically refined.

4. Computational Methods

The molecular structure calculations were performed with density functional theory (DFT) using the Gaussian16 program package [S2]. The functional M06-2X and ωB97X-D were used with the 6-31+G* basis set [S3-S6].

5. Synthetic methods and characterization

Compounds 9AnGlyEt, 9AnB, 9AnPh, and 9,10-AnB were synthesized as the following Scheme S1.

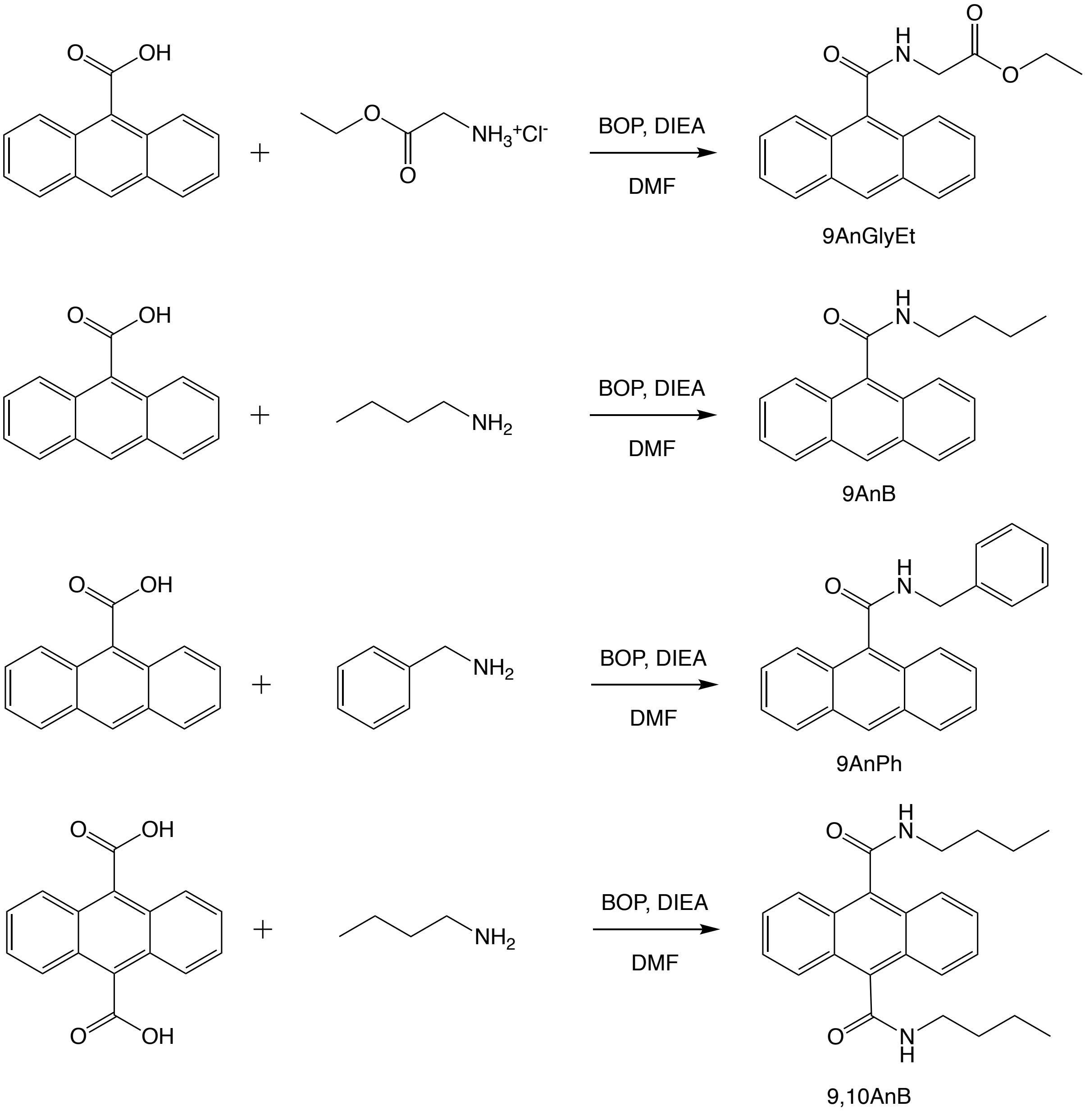

Scheme S1 Syntheses of compounds used in this study.

5-1. Synthesis of ethyl(anthracene-9-carbonyl)glycinate (9AnGlyEt) [S7]

9-Anthracenecarboxylic acid 0.042 g (1.9 x 10^{-4} mol, 1 eq) was dissolved in 7.5 mL of dehydrated DMF and stirred in a 200 mL flask. 0.24 g (5.4 x 10^{-4} mol, 3 eq) of BOP reagent was dissolved in 4.0 mL of dehydrated DMF and added dropwise. DIEA 0.093 mL (5.4 x 10^{-4} mol, 3 eq) was then dissolved in 4.0 mL of dehydrated DMF and slowly added dropwise. After stirring at room temperature for 1 h, 0.053 g (3.8 x 10^{-4} mol, 2 eq) of glycine ethyl ester hydrochloride dissolved in 6.5 mL of dehydrated DMF was slowly added dropwise. Then 0.093 mL (5.4 x 10^{-4} mol, 3 eq) of DIEA dissolved in 4.0 mL of dehydrated DMF was added dropwise and the mixture was stirred for 12h at room temperature. DMF was then distilled off, and the mixture was washed with 50 mL of DCM and 50 mL of distilled water. The solvent was removed under reduced pressure using an evaporator and the resulting solid was purified on a column (φ 3 cm x 19 cm) using Wako Gel C-200. The eluents were separated using DCM and DCM:MeOH = 300:1 and 200:1 mixtures. The solvent was removed under reduced pressure and 9AnGlyEt was reprecipitated with hexane and DCM. The precipitate was collected on a membrane filter. 9AnGlyEt was recrystallized from hexane and ethyl acetate to give yellow rhombic crystals. Yield 6.6 mg (11%).

ESI-MS (CH_3OH, *m/z*) 330.17 ($[M+Na]^+$ requires 330.11). ^1H NMR (400 MHz, $CDCl_3$, 298 K) : δ/ppm = 1.38 (tri, 3H, J = 7.2 Hz, -CH_2CH_3), 4.32 (q, 2H, J = 7.2 Hz, -OCH_2CH_3), 4.47 (d, 2H, J = 5.6 Hz, -$NHCH_2CO$-), 6.52 (s, 1H, -$CONHCH_2$-), 7.52 (m, 4H, anthracene-*H*), 8.02 (d, 2H, J = 8.4 Hz, anthracene-*H*), 8.19 (d, 2H, J = 8.4 Hz, anthracene-*H*), 8.51 (s, 1H, anthracene-*H*). ^{13}C NMR (100 MHz, $CDCl_3$, 289 K) : δ/ppm = 14.37 (-CH_2CH_3), 41.98 (-OCH_2CH_3), 61.91 (-$NHCH_2C$(=O)-), 125.26 (anthracene-*C*), 125.70 (anthracene-*C*), 126.97 (anthracene -*C*), 128.32 (anthracene -*C*), 128.62 (anthracene -*C*), 128.74 (anthracene -*C*), 131.06 (anthracene -*C*), 131.17 (anthracene -*C*), 169.81 (anthracene -*C*(=O)NH-), 169.96 (-CH_2C(=O)O-). mp 155-156 °C. Anal. Calcd. for $C_{19}H_{17}NO_3$: C, 74.25; H, 5.57; N, 4.55%. Found : C, 74.19; H, 5.56; N, 4.38%.

^{1}H NMR (400 MHz, $CDCl_3$, 298 K)

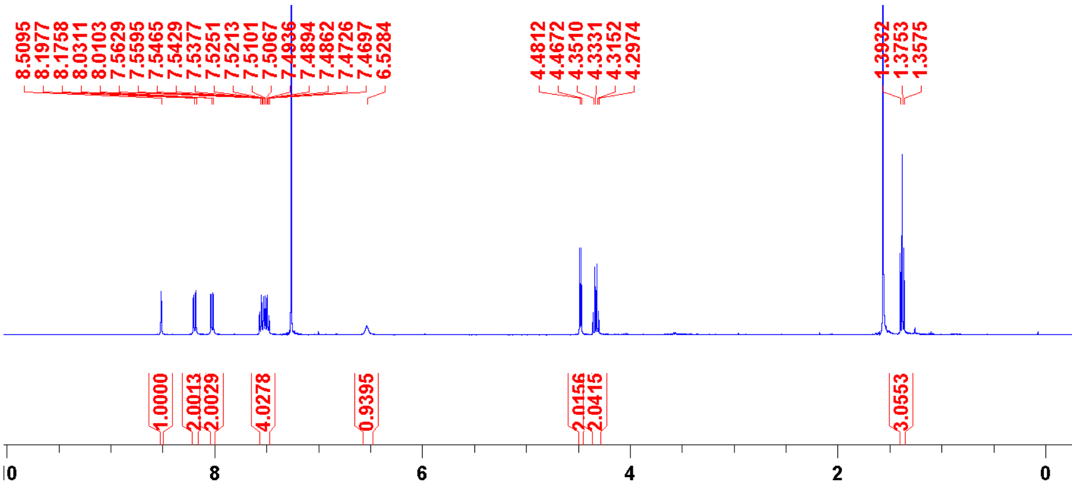

^{13}C NMR (100 MHz, $CDCl_3$, 289 K)

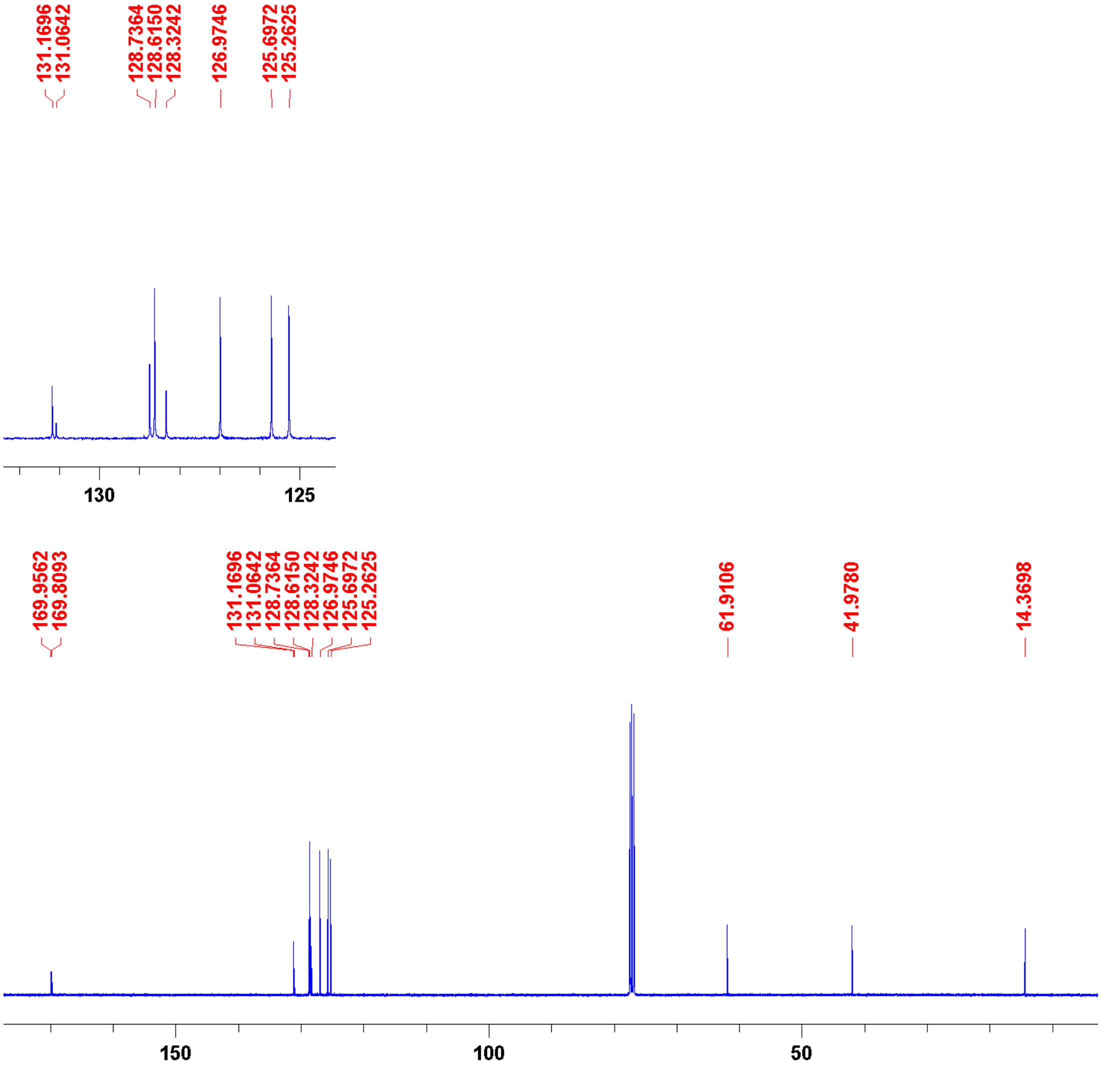

Figure S1 ^{1}H and ^{13}C NMR of 9AnGlyEt.

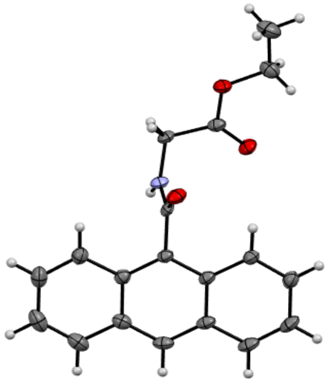

Figure S2 ORTEP diagram of 9AnGlyEt.

Table S1 Crystallographic data of 9AnGlyEt.

Formula	$C_{19}H_{17}NO_3$
Deposition Number (CCDC)	2149833
Formula Weight	307.35
Crystal system	triclinic
Space group	P-1
a (Å)	8.9450
b (Å)	9.2434
c (Å)	10.47923
α (deg)	111.862
β (deg)	93.627
γ (deg)	103.670
**Z**	2
λ (Cu Kα)	1.54187
θ max	73.91
μ (cm^{-1})	7.29
Dc (Mgm^{-3})	1.078
T (K)	296
V ($Å^3$)	770.29
R_1	0.0529
R_w	0.1573

5-2. Synthesis of *N*-butylanthracene-9-carboxamide (9AnB)

0.040 g (1.8 x 10^{-4} mol) of 9-anthracenecarboxylic acid was dissolved in 7.5 mL of dehydrated DMF. To this was added dropwise 0.24 g (5.4 x 10^{-4} mol, 3 eq) of BOP reagent dissolved in 4.0 mL of dehydrated DMF. DIEA 0.093 mL (5.4 x 10^{-4} mol, 3 eq) dissolved in 4.0 mL of dehydrated DMF was then added dropwise. After stirring at room temperature for 1 h, *n*-butylamine 0.036 mL (3.6 x 10^{-4} mol, 2 eq) dissolved in 6.5 mL of dehydrated DMF was added dropwise. Then 0.093 mL (5.4 x 10^{-4} mol, 3 eq) of DIEA dissolved in 4.0 mL of dehydrated DMF was added dropwise and stirred for 12h at room temperature. After the stirring was completed, DMF was distilled off, and the mixture was washed with 50 mL of DCM and 50 mL of distilled water. The collected organic layer was dried in an evaporator and the residue was purified on a column (*φ* 3 cm x 17 cm) using Wako Gel C-200. The eluents were separated using DCM and DCM:MeOH = 300:1 and 200:1 mixtures. The solvent was removed under reduced pressure and 9AnB was recrystallized from MeOH to give colorless plate-like crystals. Yield : 0.024 g (48%).

ESI-MS (CH_3OH, *m/z*) 299.96 ($[M+Na]^+$ requires 300.14). ^1H NMR (400 MHz, CD_3OD, 298 K, TMS) : δ/ppm = 1.00 (tri, 3H, J = 7.4 Hz, $-CH_2CH_3$), 1.50 (sext, 2H, J = 7.4 Hz, $-CH_2CH_2CH_3$), 1.71 (quin, 2H, J = 7.4 Hz, $-CH_2CH_2CH_2-$), 3.58 (tri, 2H, J = 6.7 Hz, $-NHCH_2CH_2-$), 7.48 (m, 4H, anthracene-*H*), 7.96 (d, 2H, J = 7.7 Hz, anthracene-*H*), 8.03 (d 2H, anthracene-*H*), 8.53 (s, 1H, anthracene-*H*). ^{13}C NMR (100 MHz, $CDCl_3$, 289 K) : δ/ppm = 13.90 ($-CH_2\mathit{CH_3}$), 20.35 ($-CH_2\mathit{CH_2}CH_3$), 31.87 ($-CH_2\mathit{CH_2}CH_2-$), 40.04 ($-NH\mathit{CH_2}CH_2-$), 125.18 (anthracene-*C*), 125.60 (anthracene-*C*), 126.78 (anthracene -*C*), 128.12 (anthracene -*C*), 128.26 (anthracene -*C*), 128.62 (anthracene -*C*), 131.20 (anthracene -*C*), 131.23 (anthracene -*C*), 169.59 (anthracene -*C*(=O)NH-). mp 178-179 °C. Anal. Calcd. for $C_{19}H_{20}NO$: C, 82.30; H, 6.90; N, 5.10%. Found : C, 82.19; H, 6.88; N, 4.98%.

^{1}H NMR (400 MHz, CD_3OD, 298 K, TMS)

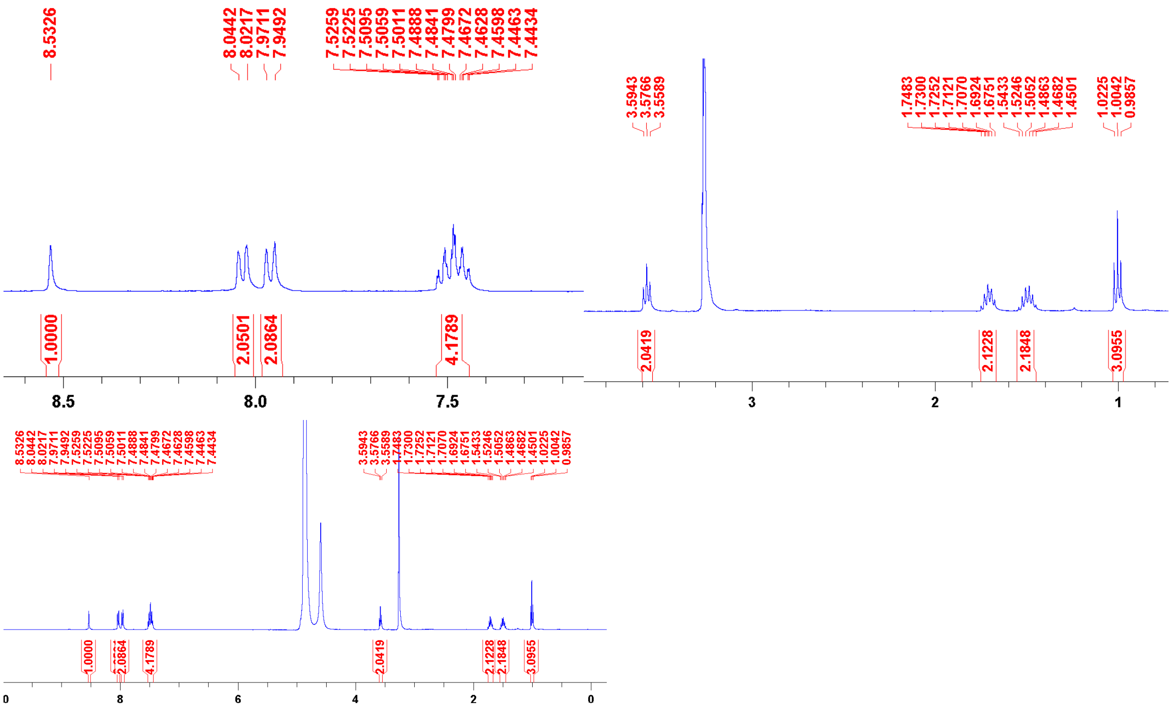

^{13}C NMR (100 MHz, $CDCl_3$, 289 K)

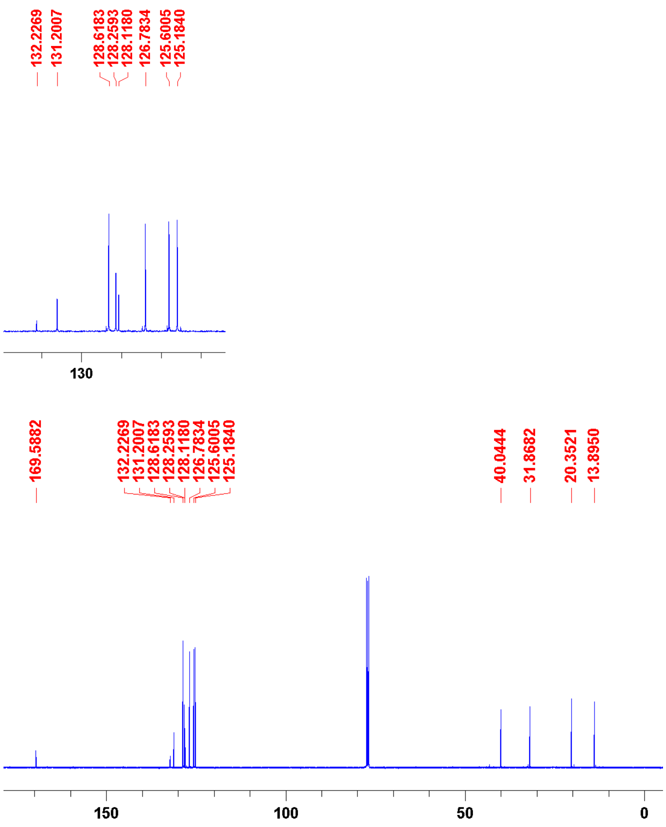

Figure S3 ^{1}H and ^{13}C NMR of 9AnB.

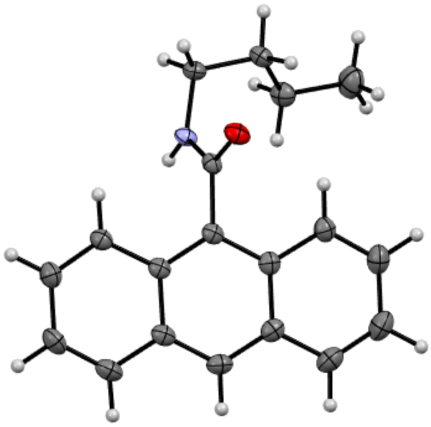

Figure S4 ORTEP diagram of 9AnB.

Table S2 Crystallographic data of 9AnB.

Formula	$C_{19}H_{19}NO$
Deposition Number (CCDC)	2149831
Formula Weight	277.37
Crystal system	orthorhombic
Space group	Pbca
a (Å)	9.74760(16)
b (Å)	9.04653(15)
c (Å)	34.1643(7)
α (deg)	90.0000
β (deg)	90.0000
γ (deg)	90.0000
**Z**	8
λ (Cu Kα)	1.54184
θ max	74.250
μ (cm^{-1})	5.84
Dc (Mgm^{-3})	1.223
T (K)	123
V ($Å^3$)	3012.67(9)
R_1	0.0410
R_w	0.1126

5-3. Synthesis of *N*-benzylanthracene-9-carboxamide (9AnPh)

0.040 g (1.8 x 10^{-4} mol) of 9-anthracenecarboxylic acid was dissolved in 7.5 mL of dehydrated DMF and stirred. 0.24 g (5.4 x 10^{-4} mol, 3 eq) of BOP reagent dissolved in 4.0 mL of dehydrated DMF was added dropwise. DIEA 0.093 mL (5.4 x 10^{-4} mol, 3 eq) dissolved in 4.0 mL of dehydrated DMF was then added dropwise. After 2 h of stirring at room temperature, benzylamine 0.039 mL (3.6 x 10^{-4} mol, 2 eq) dissolved in 7.0 mL of dehydrated DMF was added dropwise. Then 0.093 mL (5.4 x 10^{-4} mol, 3 eq) of DIEA dissolved in 4.0 mL of dehydrated DMF was added dropwise and stirred for 12h at room temperature. After the stirring was completed, DMF was distilled off, and the mixture was washed with 50 mL of DCM and 50 mL of distilled water. The solvent was removed under reduced pressure using an evaporator and dried in vacuum. The product was purified on a column (*φ* 3 cm x 17 cm) using Wako Gel C-200. The eluents were separated using DCM and DCM:MeOH = 100:1 mixture. The solvent was removed under reduced pressure and the residue was reprecipitated with hexane and DCM. The precipitate was collected on a membrane filter. 9AnPh was recrystallized from MeOH and distilled water to give colorless needle-like crystals. Yield : 0.041 g (74%).

ESI-MS (CH_3OH, *m/z*) 333.87 ($[M+Na]^+$ requires 334.12). ^1H NMR (400 MHz, $CDCl_3$, 298 K) : δ/ppm = 4.91 (d, 2H, J = 5.7 Hz, -NHCH_2Ph), 6.30 (br, 1H, -C(=O)N*H*CH_2-), 7.32 (d, 1H, J = 7.6 Hz, phenyl-*H*), 7.39 (tri, 2H, J = 7.8 Hz, phenyl-*H*), 7.50 (m, 6H, phenyl-*H*, anthracene-*H*), 8.02 (d, 4H, J = 8.0 Hz, anthracene-*H*), 8.09 (d, 2H, J = 8.7 Hz, anthracene-*H*), 8.48 (s, 1H, anthracene-*H*). ^{13}C NMR (100 MHz, $CDCl_3$, 289 K) : δ/ppm = 44.48 (-NHCH_2Ph), 125.11 (anthracene-*C*), 125.63 (anthracene-*C*), 126.90 (anthracene -*C*), 127.93 (anthracene -*C*), 128.19 (anthracene -*C*), 128.24 (Ar-*C* in benzene), 128.50 (Ar-*C* in benzene), 128.67 (anthracene -*C*), 129.03 (Ar-*C* in benzene), 131.18 (anthracene -*C*), 131.68 (anthracene -*C*), 137.93 (Ar-*C* in benzene), 169.51 (anthracene -*C*(=O)NH-). mp 157-158 °C. Anal. Calcd. for $C_{27}H_{17}NO$: C, 84.90; H, 5.50; N, 4.50%. Found : C, 84.57; H, 5.80; N, 4.38%.

^{1}H NMR (400 MHz, $CDCl_3$, 298 K)

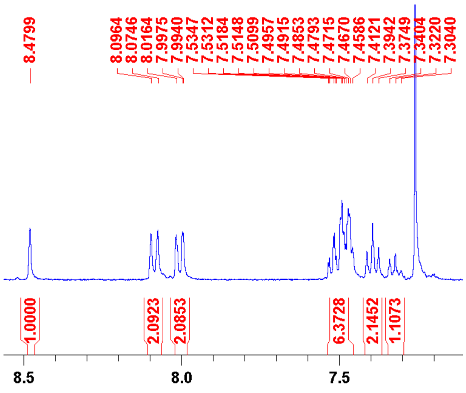

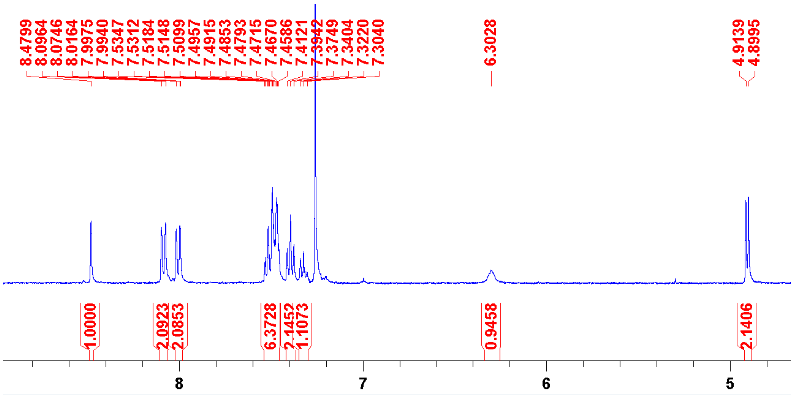

^{13}C NMR (100 MHz, $CDCl_3$, 289 K)

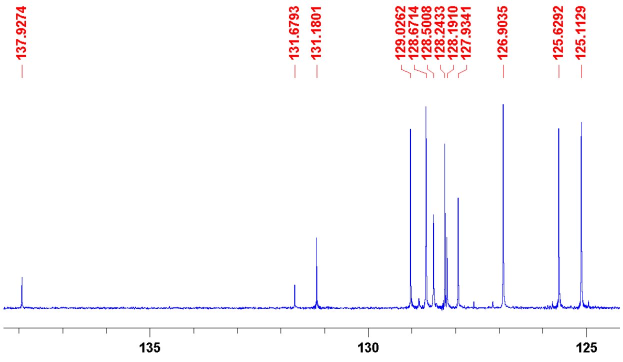

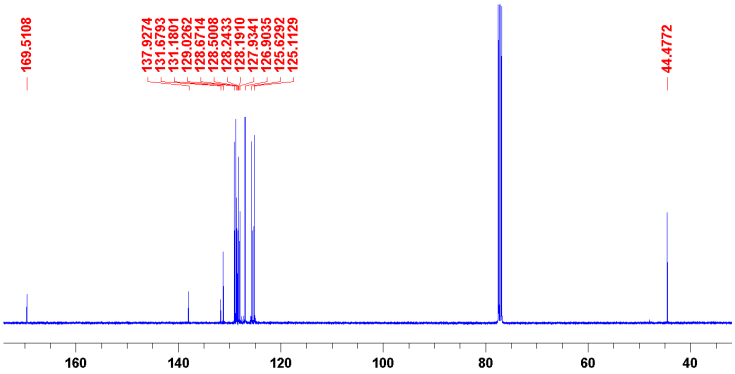

Figure S5 ^{1}H and ^{13}C NMR of 9AnPh.

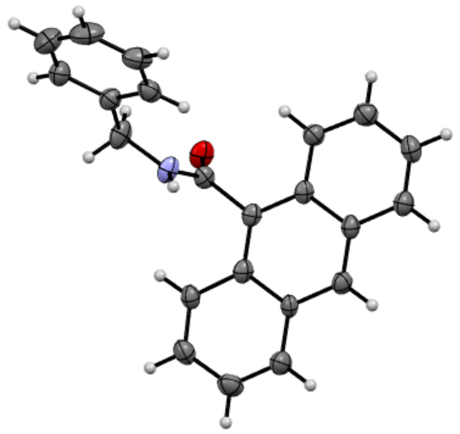

Figure S6 ORTEP diagram of 9AnPh.

Table S3 Crystallographic data of 9AnPh.

Formula	$C_{22}H_{17}NO$
Deposition Number (CCDC)	2149839
Formula Weight	311.37
Crystal system	triclinic
Space group	P-1
a (Å)	9.0368(5)
b (Å)	15.2490(7)
c (Å)	24.7924(6)
α (deg)	90.484
β (deg)	93.805
γ (deg)	90.777
**Z**	2
λ (Cu Kα)	1.54184
θ max	73.5730
μ (cm^{-1})	0.579
Dc (Mgm^{-3})	1.214
T (K)	123
V ($Å^3$)	3408.5(2)
R_1	0.1618
R_w	0.4055

5-4. Synthesis of *N*[9], *N*[10]-dibutylanthracene-9,10-dicarboxamide (9,10AnB)

0.048 g (1.8 x 10^{-4} mol) of 9,10-anthracenedicarboxylic acid was dissolved in 7.5 mL of dehydrated DMF and stirred in a 200 mL flask. 0.48 g (1.1 x 10^{-3} mol, 6 eq) of BOP reagent was dissolved in 8.0 mL of dehydrated DMF and dropped. DIEA 0.19 mL (1.1 x 10^{-3} mol, 6 eq) was then dissolved in 8.0 mL of dehydrated DMF and added slowly dropwise. After stirring at room temperature for 1h, butylamine 0.072 mL (7.3 x 10^{-4} mol, 4 eq) dissolved in 15 mL of dehydrated DMF was slowly added dropwise. Then 0.14 mL (8.1 x 10^{-4} mol, 4 eq) of DIEA dissolved in 15 mL of dehydrated DMF was added dropwise and stirred at room temperature for 12h. DMF was distilled off, and the mixture was washed with 50 mL of DCM and 50 mL of distilled water. The solvent was removed under reduced pressure using an evaporator and dried in vacuum. The product was purified on a column (*φ* 3 cm x 17 cm) using Wako Gel C-200. The eluents were separated using DCM and DCM:MeOH = 100:1 and 200:3 mixtures. The solvent was removed under reduced pressure and 9,10AnB was recrystallized from MeOH and hexane to give colorless thin plate crystals. Yield : 23 mg (33%).

ESI-MS (CH_3OH, *m/z*) 377.29 ($[M+H]^+$ requires 377.22). ^1H NMR (400 MHz, CD_3OD, 298 K, TMS) : δ/ppm = 1.06 (tri, 6H, J = 7.4 Hz, -CH_2CH_3), 1.55 (sext, 4H, J = 7.2 Hz, -$CH_2CH_2CH_3$), 1.77 (quin, 4H, J = 7.3 Hz, -$CH_2CH_2CH_2$-), 3.63 (tri, 4H, J = 7.1 Hz, -$NHCH_2CH_2$-), 7.58 (dd, 4H, anthracene-*H*), 8.04 (dd, 4H, anthracene-*H*). ^{13}C NMR (100 MHz, CD_3OD, 289 K) : δ/ppm = 14.96 (-$CH_2\mathit{CH_3}$), 22.21 (-$CH_2\mathit{CH_2}CH_3$), 33.34 (-$CH_2\mathit{CH_2}CH_2$-), 41.69 (-$NH\mathit{CH_2}CH_2$-), 127.29 (anthracene-*C*), 128.51 (anthracene-*C*), 129.52 (anthracene -*C*), 135.88 (anthracene -*C*), 172.52 (anthracene -*C*(=O)NH-). mp 207-208 °C. Anal. Calcd. for $C_{24}H_{28}N_2O_2$: C, 76.56; H, 7.50; N, 7.44%. Found : C, 76.60; H, 7.56; N, 7.34%.

^{1}H NMR (400 MHz, CD_3OD, 298 K, TMS)

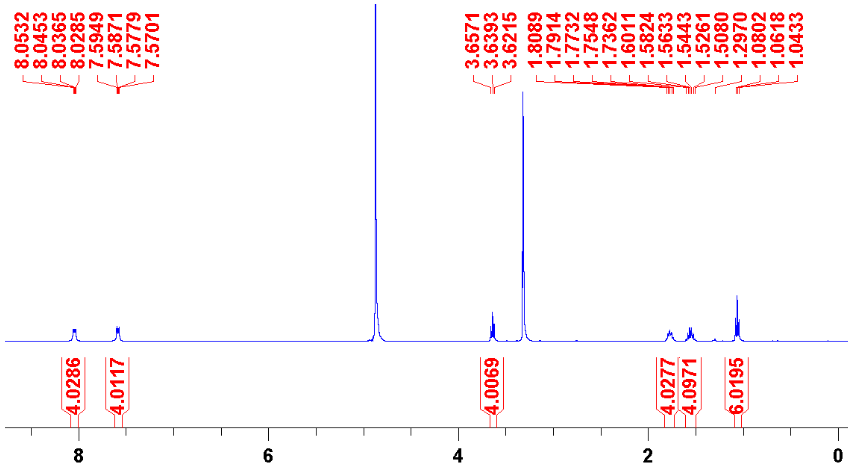

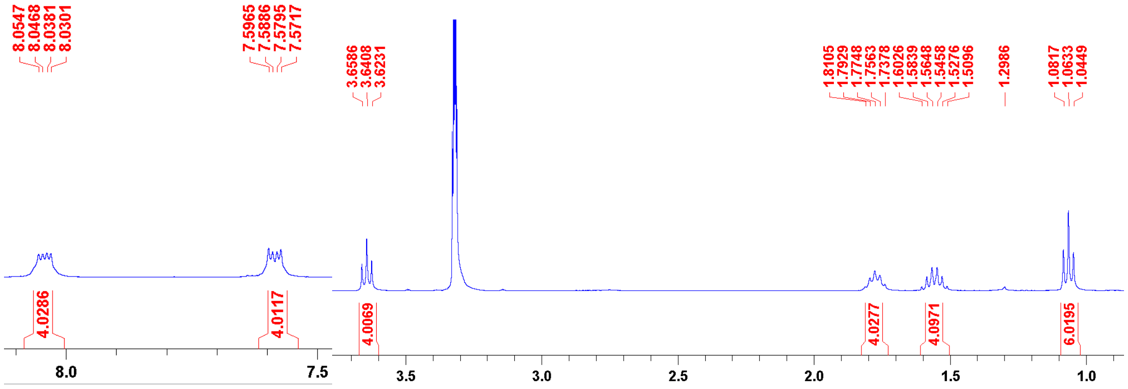

^{13}C NMR (100 MHz, CD_3OD, 289 K)

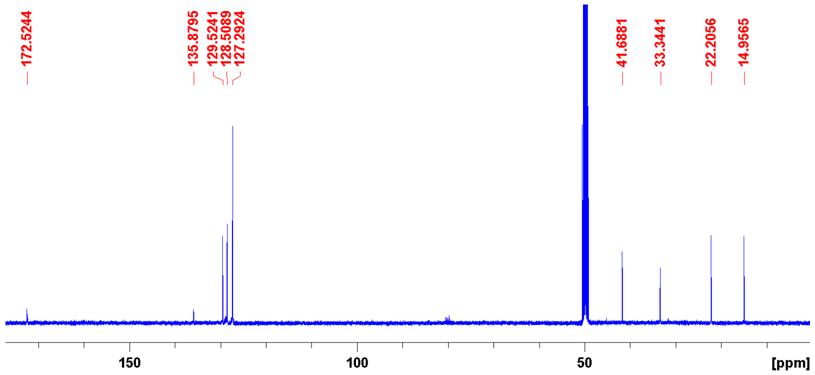

Figure S7 ^{1}H and ^{13}C NMR of 9,10AnB.

6. Fluorescent color change

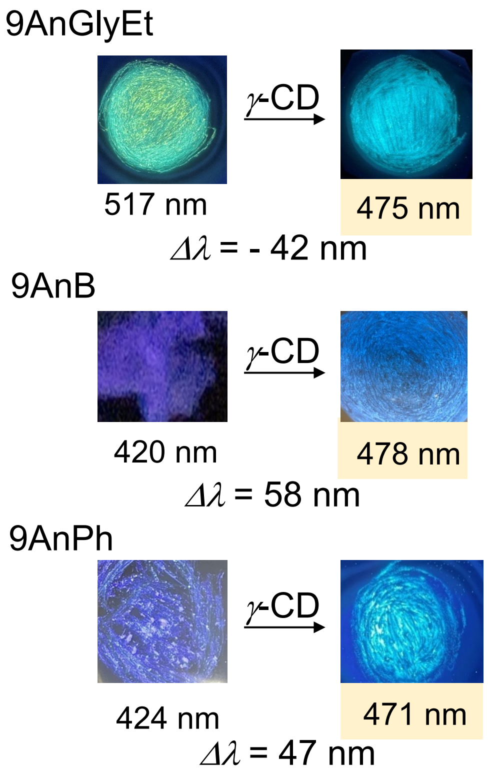

Figure S8 Photographs of anthracene derivatives (left) and their γ-CD complexes (right) under the irradiation of 365 nm.

7. Circular dichroism spectra

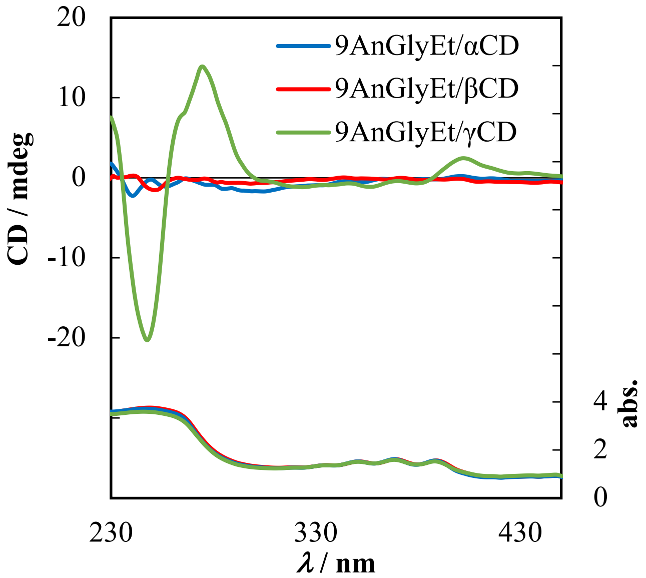

Figure S9 Circular dichroism (upper panel) and UV-vis (lower panel) spectra of 9AnGlyEt/*α*-CD, 9AnGlyEt/*β*-CD and 9AnGlyEt/*γ*-CD in KBr pellet at room temperature.

8. ^{13}C CP/MAS NMR

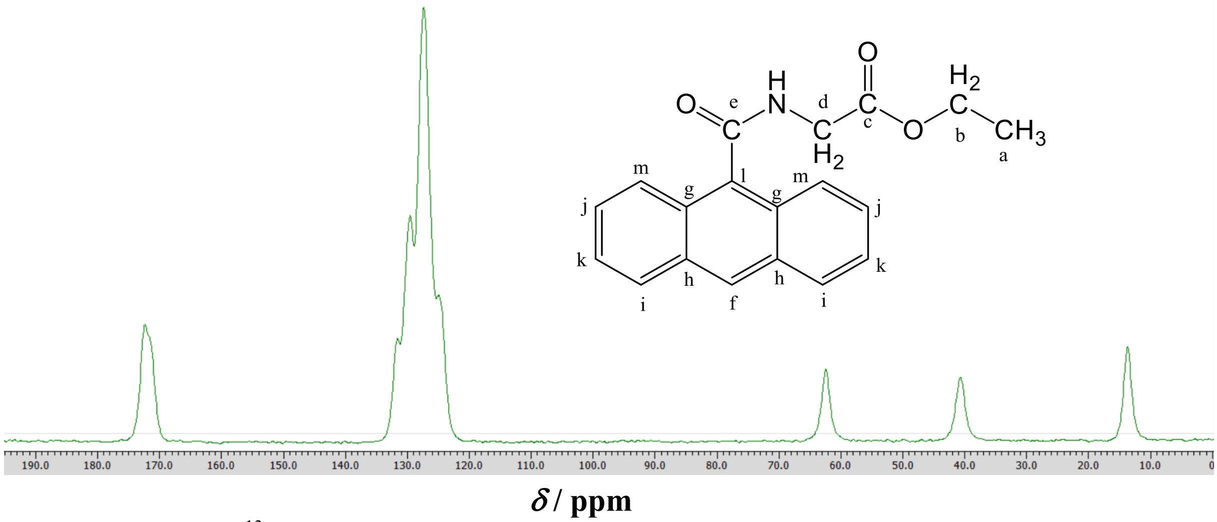

Figure S10 ^{13}C CP/MAS NMR (100.53 MHz) spectrum of 9AnGlyEt in solid state.

Table S4 Assignment of ^{13}C NMR spectrum of 9AnGlyEt in solid state.

Chemical Shift (δ / ppm) (J / Hz)	Assignment
13.70	a
40.63	b
62.42	d
124.91~131.58	f, g, h, i, j, k, l, m
172.41	e, c

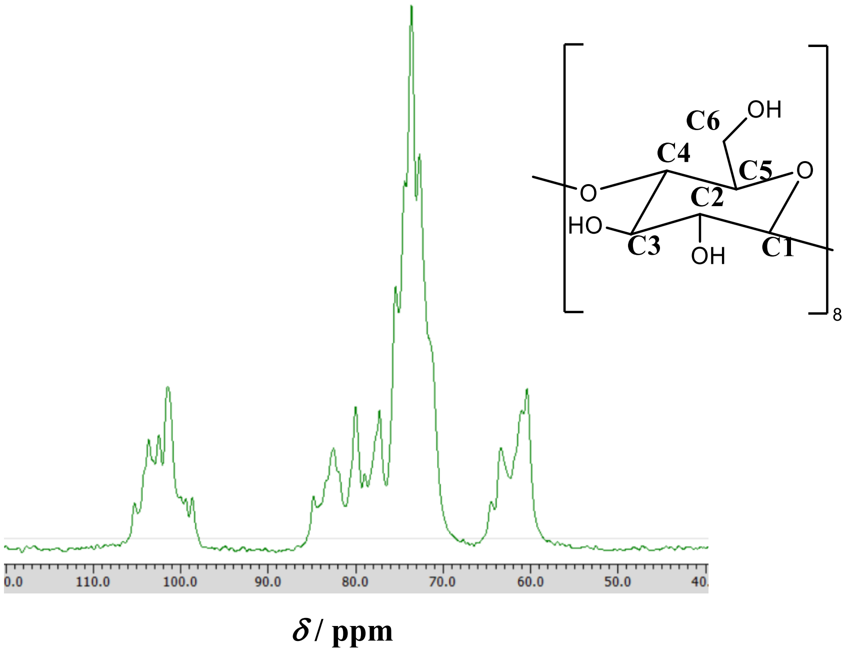

Figure S11 ^{13}C CP/MAS NMR (100.53 MHz) spectrum of *γ*-CD in solid state.

Table S5 Assignment of ^{13}C NMR spectrum of *γ*-CD in solid state.

Chemical Shift (*δ* / ppm) (*J* / Hz)	Assignment
60.41 ~ 63.41	C6
72.41 ~ 77.24	C2, C3, C5
79.80 ~ 84.77	C4
99.46 ~ 103.68	C1

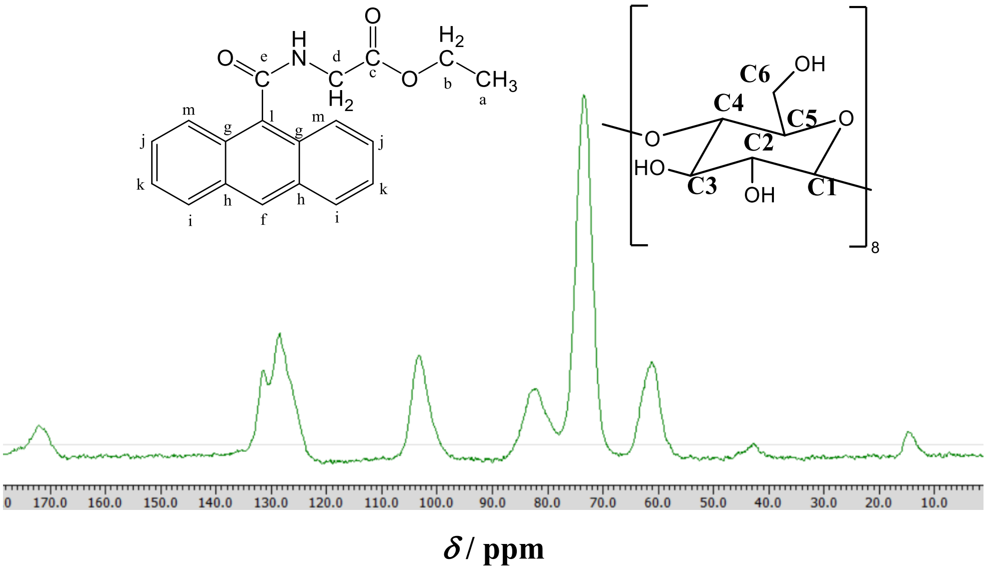

Figure S12 ^{13}C CP/MAS NMR (100.53 MHz) spectrum of 9AnGlyEt / *γ*-CD in solid state.

Table S6 Assignment of ^{13}C NMR spectrum of 9AnGlyEt and *γ*-CD in solid state.

Chemical Shift (*δ* / ppm) (*J* / Hz)	Assignment
14.49	a
42.71	b
61.68	d, C6
73.43	C2, C3, C5
82.43	C4
103.17	C1
128.51~131.45	f, g, h, i, j, k, l, m
172.08	e, c

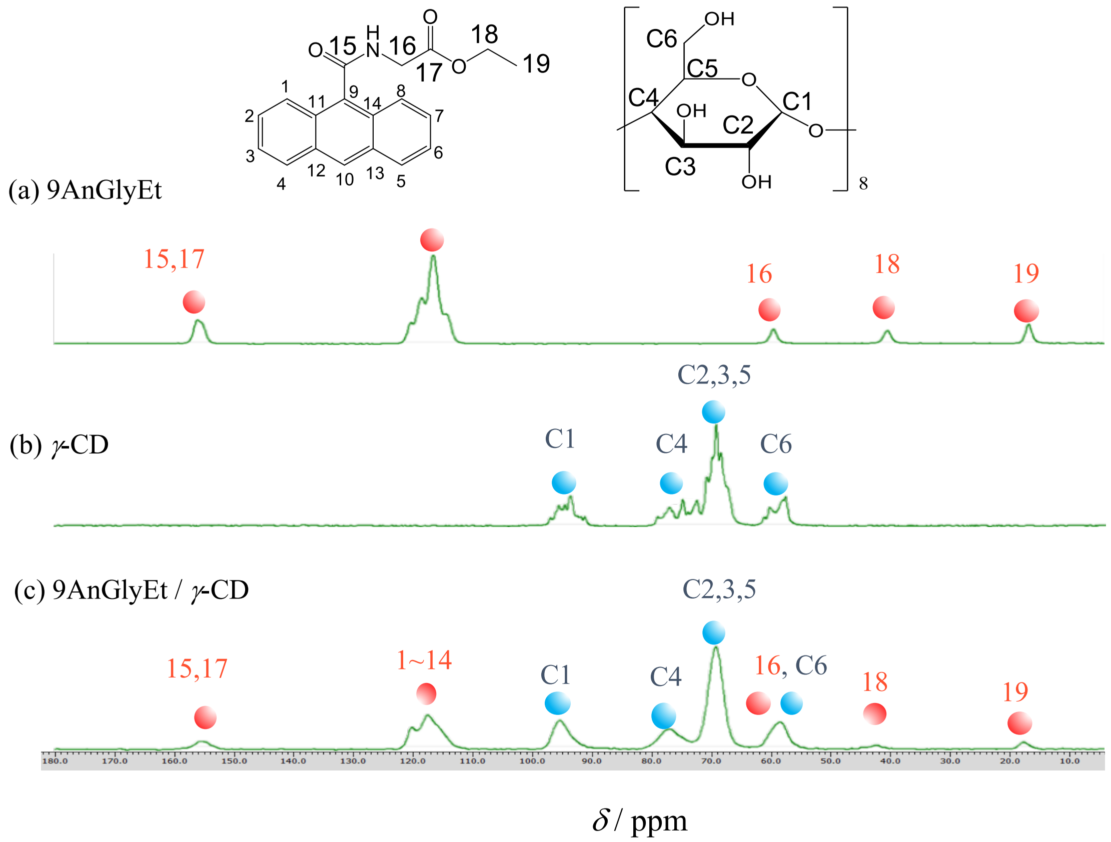

Figure S13 ^{13}C CP/MAS NMR (100.53 MHz) spectrum of (a) 9AnGlyEt, (b) γ-CD and (c) 9AnGlyEt / γ-CD in solid state.

9. Reference

S1. SHELXL 97, Sheldrick, G. M. University of Göttingen, Germany (1997).

S2. Gaussian 16, Revision D.01, Frisch, M. J., Trucks, G. W., Schlegel, H. B., Scuseria, G. E., Robb, M. A., Cheeseman, J. R., Scalmani, G., Barone, V., Petersson, G. A., Nakatsuji, H., Li, X., Caricato, M., Marenich, A., Bloino, J., Janesko, B. G., Gomperts, R., Mennucci, B., Hratchian, H. P., Ortiz, J. V., Izmaylov, A. F., Sonnenberg, J. L., Williams-Young, D., Ding, F., Lipparini, F., Egidi, F., Goings, J., Peng, B., Petrone, A., Henderson, T., Ranasinghe, D., Zakrzewski, V. G., Gao, J., Rega, N., Zheng, G., Liang, W., Hada, M., Ehara, M., Toyota, K., Fukuda, R., Hasegawa, J., Ishida, M., Nakajima, T., Honda, Y., Kitao, O., Nakai, H., Vreven, T., Throssell, K., Montgomery, Jr., J. A., Peralta, J. E., Ogliaro, F., Bearpark, M., Heyd, J. J., Brothers, E., Kudin, K. N., Staroverov, V. N., Keith, T., Kobayashi, R., Normand, J., Raghavachari, K., Rendell, A., Burant, J. C., Iyengar, S. S., Tomasi, J., Cossi, M., Millam, J. M., Klene, M., Adamo, C., Cammi, R., Ochterski, J. W., Martin, R. L., Morokuma, K., Farkas, O., Foresman, J. B., Fox, D. J.: Gaussian, Inc., Wallingford CT (2016).

S3. Adamo, C., Jacquemin, D.: The calculations of excited-state properties with time-dependent density functional theory, Chem. Soc. Rev. **42**, 845-856 (2013).

S4. Zhao, Y., Truhlar, D. G.: The M06 suite of density functionals for main group thermochemistry, thermochemical kinetics, noncovalent interactions, excited states, and transition elements: two new functionals and systematic testing of four M06-class functionals and 12 other functionals, Theor. Chem. Acc. **120**, 215-241 (2008).

S5. Chai, J.-D., Head-Gordon, M.: Long-range corrected hybrid density functionals with damped atom-atom dispersion corrections, Phys. Chem. Chem. Phys. **10**, 6615-6620 (2008).

S6. Gao, Y., Yang, B.: Excimer formation and evolution of excited state properties in discrete dimeric stacking of an anthracene derivative: a computational investigation, Phys. Chem. Chem. Phys., **20**, 12129-12137 (2018).

S7. Sawai, M., Matsumoto, S., Mimura, Y., Imai, Y., Yamazaki, S., Kanehisa, N., Tohnai, N., Nakata, E., Takashima, H.: Circularly Polarized Luminescence (CPL) Characteristics of Hydrophobic Pyrene Derivatives/*γ*-Cyclodextrin (*γ*-CD) Complexes in Aqueous Solution Dissolved by Grinding, J. Incl. Phenom. Macrocycl. Chem., **102**, 133-142 (2022).
